# Supplementary material for: Predicting visual function by interpreting a neuronal wiring diagram
Source: Nature. 2024 Oct 2;634(8032):113–23. doi: 10.1038/s41586-024-07953-5 (PMC11446822; doi:10.1038/s41586-024-07953-5)
Supplement: Supplementary file 6 — CRF and ERF predictions for individual TmY4 and TmY9 cells. Analogous to Supplementary Data 3, but for TmY target types. Shown are the top four monosynaptic pathways, the strongest pathway passing through each of the top ten intermediary types (ranking from Extended Data Fig. 7), and the trisynaptic pathway Tm1–TmY–Dm3–TmY (see the section entitled Prediction of spatial normalization). [file 41586_2024_7953_MOESM6_ESM.zip › DataS4/TmY9q/720575940633752927.pdf]

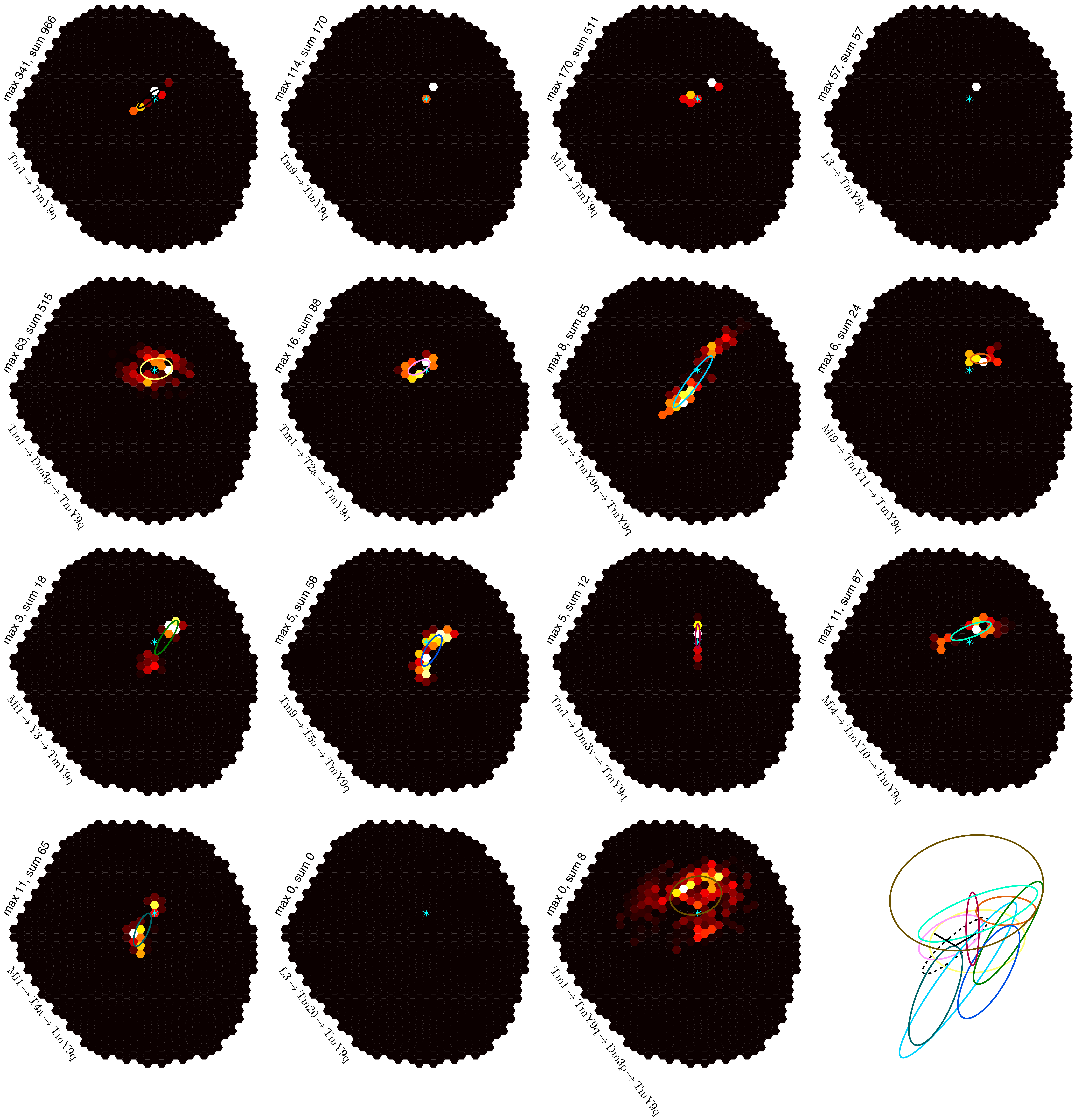

max 341, sum 966
Tm1 → TmY9q
max 114, sum 170
Tm9 → TmY9q
max 170, sum 511
Mi1 → TmY9q
max 57, sum 57
L3 → TmY9q
max 63, sum 515
Tm1 → Dm3p → TmY9q
max 16, sum 88
Tm1 → T2a → TmY9q
max 8, sum 85
Tm1 → TmY9q → TmY9q
max 6, sum 24
Mi9 → TmY11 → TmY9q
max 3, sum 18
Mi1 → Y3 → TmY9q
max 5, sum 58
Tm9 → T5a → TmY9q
max 5, sum 12
Tm1 → Dm3v → TmY9q
max 11, sum 67
Mi4 → TmY10 → TmY9q
max 11, sum 65
Mi1 → T4a → TmY9q
max 0, sum 0
L3 → Tm20 → TmY9q
max 0, sum 8
Tm1 → TmY9q → Dm3p → TmY9q
